# Imaging of human papilloma virus (HPV) related oropharynx tumour: what we know to date

**DOI:** 10.1186/s13027-023-00530-x

**Published:** 2023-10-09

**Authors:** Eleonora Bicci, Leonardo Calamandrei, Francesco Mungai, Vincenza Granata, Roberta Fusco, Federica De Muzio, Luigi Bonasera, Vittorio Miele

**Affiliations:** 1 Department of Radiology, University of Florence - Azienda Ospedaliero-Universitaria Careggi, Florence, 50134 Italy; 2 Department of Experimental and Clinical Biomedical Sciences, Radiodiagnostic Unit n. 2, University of Florence - Azienda Ospedaliero-Universitaria Careggi, Florence, 50134 Italy; 3 Division of Radiology, Istituto Nazionale Tumori IRCCS Fondazione Pascale-IRCCS di Napoli, Naples, 80131 Italy; 4 Medical Oncology Division, Igea SpA, Naples, 80013 Italy; 5 Italian Society of Medical and Interventional Radiology (SIRM), SIRM Foundation, Milan, 20122 Italy; 6 Department of Medicine and Health Sciences V. Tiberio, University of Molise, Campobasso, 86100 Italy

**Keywords:** Oropharynx, HPV, Head and Neck, Oncology, Magnetic resonance imaging, Ultrasound, Computed tomography, Radiomics

## Abstract

The tumours of head and neck district are around 3% of all malignancies and squamous cell carcinoma is the most frequent histotype, with rapid increase during the last two decades because of the increment of the infection due to human papilloma virus (HPV). Even if the gold standard for the diagnosis is histological examination, including the detection of viral DNA and transcription products, imaging plays a fundamental role in the detection and staging of HPV + tumours, in order to assess the primary tumour, to establish the extent of disease and for follow-up. The main diagnostic tools are Computed Tomography (CT), Positron Emission Tomography-Computed Tomography (PET-CT) and Magnetic Resonance Imaging (MRI), but also Ultrasound (US) and the use of innovative techniques such as Radiomics have an important role. Aim of our review is to illustrate the main imaging features of HPV + tumours of the oropharynx, in US, CT and MRI imaging. In particular, we will outline the main limitations and strengths of the various imaging techniques, the main uses in the diagnosis, staging and follow-up of disease and the fundamental differential diagnoses of this type of tumour. Finally, we will focus on the innovative technique of texture analysis, which is increasingly gaining importance as a diagnostic tool in aid of the radiologist.

## Introduction

The tumours of head and neck district are around 3% of all malignancies and squamous cell carcinoma is the most frequent histotype [1–4]. The incidence of these subtypes of tumour has been rapidly increasing during the last two decades because of the increment of the infection due to human papilloma virus (HPV), cause of tumours that mainly develop into lingual and palatine tonsils tissue [5]. Regarding HPV-positive (HPV+) squamous cell tumour, it is most commonly found in males with age of around 40–60 years. They are not related with tobacco exposure or smoking, or alcohol consumption. The most frequent genotypes involved are genotype 16 and 18, that are considered therefore oncogenic [6].

The diagnosis of OPSCC is based on pan-endoscopy with biopsies of the primary lesion [7]. When traditional endoscopic techniques might fail to identify the primitive lesion, the better visualization and freedom of motion of trans-oral robotic surgery (TORS) techniques might also help with biopsy [8, 9]. The gold standard for the diagnosis is histological examination, including the detection of viral DNA and transcription products [10].

On imaging HPV + tumours differ from HPV-negative (HPV-) ones [11]. Imaging is mandatory in order to permit the correct extension of disease, to evaluate lymph-nodal involvement and the presence of distant metastasis.

Ultrasound is used for the evaluation of suspected laterocervical lymphnodal metastasis. HPV + tumours usually show cystic and necrotic nodal metastasis, differently from HPV- ones [12]. At computed tomography (CT) and magnetic resonance imaging (MRI) HPV + primary tumours often detect well-defined lesions with exophytic growth with vivid homogeneous enhancement [12–14]. The superior soft-tissue contrast of MRI imaging allows for easier detection of nodal metastases and better definition of tumour extension, margins, and vascular or nervous involvement [15–19].

Also, innovative new techniques are also increasingly being used in the evaluation of this type of tumour, including MRI perfusion imaging and texture analysis.

The aim of this review is to illustrate the main imaging features of HPV + tumours of the oropharynx, in US, CT and MRI imaging. In particular, we will outline the main limitations and strengths of the various imaging techniques, the main uses in the diagnosis, staging and follow-up of disease and the fundamental differential diagnoses of this type of tumour. Finally, we will focus on the innovative technique of texture analysis, which is increasingly gaining importance as a diagnostic tool in aid of the radiologist.

## Diagnostic role of ultrasound

While the inherent limits of ultrasound (US) imaging don’t generally allow for the evaluation of primary site HPV-related cancer, its availability, relative low-cost and its great definition for the study of superficial structures make it ideal to investigate suspicious lymph node swelling in the head and neck district [20–23]. Thus, the main role played by US in the study of HPV-related cancer consists in the identification of potential nodal metastatic disease in tumors of unknown primary origin [11, 20–22, 24].

While no specific nodal station in related to HPV disease as is, it is true that metastatic drainage in the head and neck is district specific [21, 22, 25, 26]. Thus, a lymph node with anomalous features on ultrasound might be indicative of the presence of a primary tumor which location might be inferred by the radiologist with sufficient knowledge of the pattern of distribution of metastatic nodes.

Cancers of the oropharynx, hypopharynx and larynx tend to metastasize more frequently to the internal jugular chain (leves II-III-IV), while upper cervical and submandibular chains (levels Ia-Ib and IIa) are more frequently related to oral cancers [21, 22]. While HPV-positive nasopharyngeal carcinomas are rarer overall, it is important to note that these neoplasms tend to metastasize to the upper cervical or posterior triangle stations.

### Size

Size is considered an indirect indicator of malignancy.

Optimal cut-off values for the minimal diameter have been identified as 10 mm cervical nodes on an axial plane [27]. It is important to note that size alone cannot be used to evaluate possible metastatic lymph node, and it should also be kept in mind that upper neck nodes are usually bigger than lower neck ones.

In addition, size evaluation over time can be useful for patients that have already been diagnosed with a primary head and neck tumour.

### Shape

Normal or inflammatory nodes usually appear as oval or flat in shape. Metastatic lymph nodes tend to be round in shape. Borders of malignant nodes are typically sharper and better defined from surrounding tissues than normal on ultrasound [21, 28]. A possible explanation of this phenomenon is the increased acoustic impedance difference between node and tissue that is a byproduct of reduced fatty infiltration and the presence of tumor cells that have replaced lymphoid tissue inside the node itself [21].

### Echogenicity

**Fig. 1:**
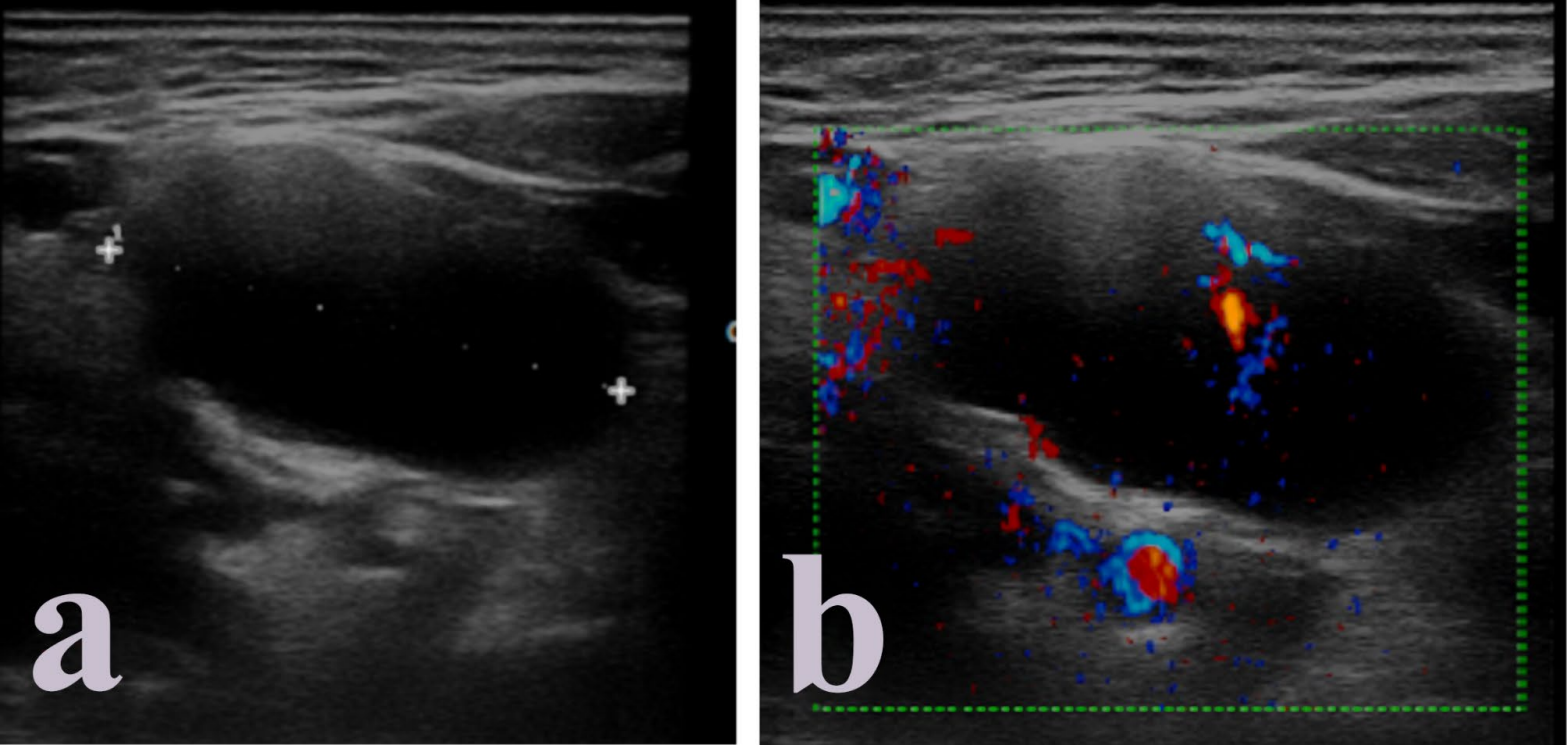
Echolucent cystic necrosis in HPV + metastatic cervical lymph node

HPV-related metastatic lymph nodes tend to be hypoechoic than adjacent muscle tissue. Usually, no hyperechoic hilus can be detected even though it might still be present in the earlier phases of the process of tumoral invasion of the node. A typical sign of squamous cell HPV-related carcinoma is the development of echolucent cystic necrosis (Fig. 1), which is a feature common to every imaging modality (Table 1). It should be noted that tubercular nodes and metastases form papillary thyroid cancer can also present cystic necrosis as a late alteration to nodal structure, as well as benign cysts of the neck such as branchial cleft cysts. Hyperechoic spots, sign of coagulation necrosis, are rare, as well as intranodal calcifications, which are more likely to appear as a consequence of chemotherapy.

**Table 1:** Elements of differential diagnosis between HPV + and HPV- oropharyngeal cancer across different imaging techniques

Imaging technique	HPV+	HPV-
**Ultrasound**		
Primary lesion	-	-
Metastatic lymph nodes	Size > 8 mm (9 mm subdigastric IIa) Round shape	
	Hypoechoic cystic necrosis	Hyperechoic spots (coagulation necrosis)- rare Intranodal calcification
**CT**		
Primary lesion	Smaller Exophytic growth Well defined borders	Bigger Ill-defined borders Grows by invading submucosa and adjacent muscle tissue Necrosis Ulceration
Metastatic lymph nodes	Cystic necrosis Well-defined borders Clustering	Extra-nodal extension Ill-defined borders Matting
**PET-CT**		
	Higher SUVmax	Lower SUVmax
**Conventional MRI**		
Primary lesion	Same elements as CT but better overall definition	
Metastatic lymph node		
**DWI**		
Primary lesion	Lower ADC and D_t_; Leptokurtic Right skewed ADC histogram	Higher ADC and D_t_; Symmetric Normally distributed ADC histogram
Metastatic lymph node		
**IVIM**	No significant difference in D*, skewness, kurtosis	
**DKI**		
**DCE-MRI**		
Primary lesion	Inconstant lower values of k_trans_	-
Metastatic lymph node		

Inflammatory edema of surrounding tissue might be present around all metastatic nodes, regardless of HPV correlation. Lymph node matting is rare.

### Color-doppler

Vascular pattern of metastatic nodes tends to be peripheral, as no clear hilar vascularization can be identified. In the presence of cystic necrosis, however, it is possible to observe avascular nodes due to vase destruction.

### Additional use

In addition to the imaging features that might help in the evaluation of nodal metastases, it is also important to remember the role played by US in ultrasound-guided fine needle aspiration cytology (FNAC) for the definitive diagnoses. The employment of ultrasound to provide more accurate information can be used both for diagnosis and follow-up.

## Diagnostic role of computed tomography

Along standard MRI, CT imaging has become one of the fundamental imaging techniques for both diagnosis and staging of head and neck cancer, regardless of HPV status.

### Diagnosis

CT plays a vital role in the definition of primary tumor in the head and neck region. To this end, CT has been employed both to better define the size and relation to contiguous tissues and structures of a suspect lesion found on clinical examination or during laryngoscopy and to study the head and neck district in search of a primary tumor of unknown origin when faced with suspicious lymph node swelling. To these ends, contrast-enhanced CT of the head and neck region is fundamental to both identify the lesion and to better evaluate its extension to surrounding tissues.

While definitive diagnosis of HPV related disease can only be proven through histology, a certain amount of imaging features can help the radiologist to infer the HPV status of a tumoral mass. In addition to that, a number of prognostic biomarkers can be evaluated during staging, allowing for a better definition of oncologic risk.

### Imaging elements of differential diagnosis of HPV-related disease on CT imaging

HPV-related cancers of the head and neck district tend to present certain features that can help the radiologist in their characterization (Table 1).

When it comes to non-contrast imaging, among morphological and topographical differences between HPV + and HPV- disease can be found the usually smaller overall size of HPV + lesions and their exophytic growth [13, 29].

**Fig. 2:**
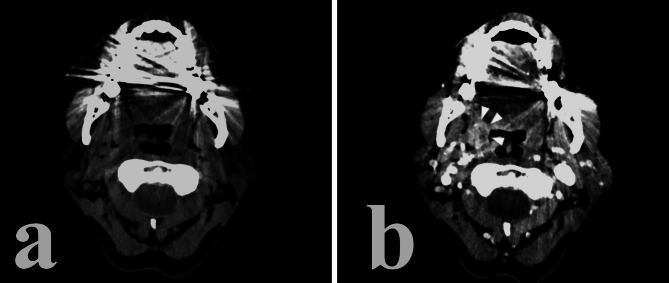
HPV + right tonsil cancer. Notice the relatively high post-contrast enhancement of the lesion, its well-defined borders and exophitic growth

**Fig. 3:**
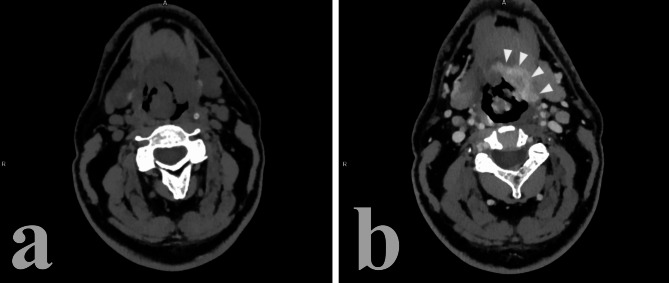
HPV- cancer of the base of the tongue and lower tonsillar pole. Notice the relatively low post-contrast enhancement, ill-defined borders and infiltration of adjacent structures such as the oral pelvis

On post-contrast CT, HPV-related cancer tends to show a stronger enhancement as opposed to HPV- disease which is usually less enhanced [30, 31] (Figs. 2 and 3). It should be noted that this difference is very subtle and not always present [13].

HPV-related cancer usually presents itself as an exophytic lesion with well-defined borders and little to no invasion of the submucosa or of the adjacent tissues, such as muscle [13, 29–32] (Fig. 2). Non-HPV lesions, on the other hand tend to show borders that are ill-defined and are more likely to develop invasion of adjacent tissue and of the submucosa [31] (Fig. 3). Ancillary features such as ulceration of the mass or necrosis can be shown in a subset of HPV + primary lesions, but they tend to be more characteristic of HPV- cancer [29, 30].

Aside from considerations that the radiologist can make on primary lesion, there are a number of features to be investigated on metastatic lymph nodes as well. Most of such considerations have already been reported in the ultrasound section of this paper and, as such, will only be cited briefly.

No specific nodal level can be correlated directly to HPV + disease, as lesions can equally metastasize to each level of the head and neck district regardless of HPV status [13, 29, 31, 33]. Nodal level involvement depends on the site of the primary tumor [21, 22]. Retro-pharyngeal metastatic nodes can be involved in both HPV + and HPV- disease equally, as happens with contralateral node involvement [29, 33].

As previously stated, cystic necrosis of lymph nodes is one of the defining features of HPV disease [21, 22, 29, 33]. Cystic necrosis can be identified on CT imaging as a regular, usually oval or circular in shape, area of hypoattenuation inside the lymph node which may show peripheral contrast enhancement on post-contrast imaging [13, 15, 34]. While lymph nodes tend to show clustering, it should be noted that in the case of HPV + disease matting of nodes is less likely to happen [29, 33].

Similarly to primary tumors, the borders of the swollen metastatic lymph node are more likely to be well-defined, with radiologic extra nodal extension (rENE) of adjacent structures being rarer in HPV + disease [13, 33].

### Staging and prognosis

CT imaging is most commonly used to stage head and neck cancer, regardless of HPV status [31, 35–37].

CT imaging of primary lesion can be used, with or without the involvement of MRI, to determine tumor size and invasion of adjacent tissue [31, 36–38]. CT can also be used to better evaluate involvement of cortical bone structures adjacent to the lesion thanks to its superior power in the definition of spatial bone and cartilage anatomy, especially when the skull base, sinuses, mandibular bone and maxilla are involved [39, 40] which is fundamental to determine possibilities of surgical approach and overall for T staging [13, 36, 37].

In addition to that, nodal involvement can be evaluated via CT in order to define N staging. While MRI can be used to detect metastatic lymph nodes, CT tends to be more reliable and, to this extent, better overall for N staging [40].

Finally, the employment of High-Resolution CT (HRCT) of the chest allows for a better evaluation of possible metastases to the lung and is thus used for M staging.

## Diagnostic role of PET-CT

Positron emission tomography-computed tomography is a technique that combines the precise anatomical definition of a CT scan with information regarding the metabolic activity of primary tumor and metastatic sites. As such PET-CT plays a fundamental role in both staging and post treatment surveillance of OPSCC, regardless of HPV status [41].

Fusion CT images produces by PET-CT may sometimes provide a better definition of primary site tumors, especially in those sites that are notoriously harder to study through other techniques (oral cavity, tongue etc.) especially with smaller lesions that might have been easy to spot on clinical examination but might be too small to show significant contrast enhancement to help differentiate from surrounding tissue [42]. This is especially useful for tumors of unknown primary site, that might be brought to the clinician’s attention through nodal involvement before the primary lesion becomes symptomatic [41, 42]. In these situations PET-CT techniques in addition to endoscopic or TORS techniques might facilitate primary site localization and have been shown to be in fact superior to CT imaging alone and comparable to DWI MRI [8, 43, 44].

When it comes to HPV-correlation, multiple studies have shown that HPV + cancers tend to present themselves with higher SUVmax values when compared to HPV- tumors [41, 45]. As such PET-CT can be another instrument to help infer HPV status in oropharyngeal cancer.

## Diagnostic role of MRI

### Conventional T1-weighted and T2-weighted MRI

#### Diagnosis

While conventional, anatomical, MRI yields results similar to CT in terms of employment in the diagnostic stage of head and neck cancer [29, 40], as well as similar findings in the differential diagnosis of HPV status, it should be noted that the arguably superior definition when it comes to soft-tissue contrast [46–48] and to possibility to employ specific sequences to better highlight anatomical structures, such as fat suppression sequences, can allow for better visualization of primary lesion anatomy and its relationship with surrounding tissue [29, 46–52].

#### Imaging elements of differential diagnosis of HPV-related disease on conventional MRI imaging

**Fig. 4:**
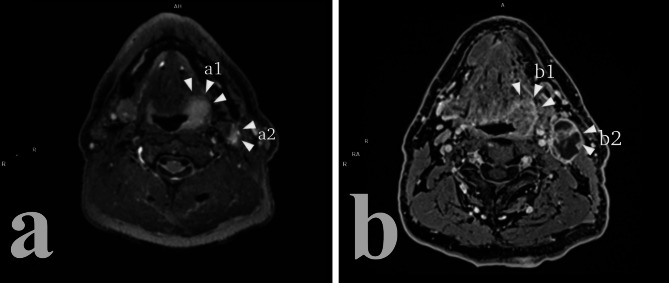
HPV + oropharyngeal cancer (a1, b1) with adjacent cystic metastatic lymph node (a2, b2). (a) T2w; (b) post-contrast T1w. The relative higher soft tissue resolution of MRI highlights the contained nature of the lesion and its well-defined borders

**Fig. 5:**
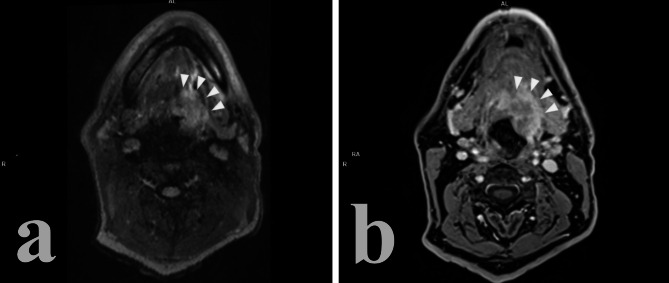
HPV- cancer of the base of the tongue. (a) T2w; (b) Post-contrast T1w. Notice the ill-defined borders and the locoregional invasion of adjacent tissue

As stated above, elements of differential diagnosis of HPV status in head and neck cancer are comparable between CT and MRI and, as a such, will only be briefly repeated (Table 1). MRI imaging of HPV + primary tumor tends to show overall smaller lesions, exophytic, with less submucosal or adjacent tissue spreading overall [13, 29, 53, 54] (Figs. 4 and 5). Ulceration and necrosis are also less common then in HPV- tumors [13, 54].

No significant difference seems to be present after contrast administration between HPV + and HPV- lesions [13, 29, 53–55].

When it comes to nodal involvement, the same consideration that were made for CT are valid for MRI, with metastasized lymph nodes that tend to develop cystic necrosis (focal area of homogenous high intensity on T2-weighted imaging with or without peripheral contrast-enhancement on post-contrast T1-weighted imaging), to form clusters without signs of matting, with well-defined borders and little to no radiologic extra-nodal extension (rENE) [13, 24, 56–58] (Fig. 4).

#### Staging and prognosis

Due to its superior accuracy and contrast resolution, MRI is often employed for the local staging of tumoral lesions, and local extension to adjacent tissues [26, 52, 59].

Locoregional nodal involvement can also be studied with MRI techniques with high sensitivity and specificity [59].

### Functional MRI

In these recent years, the employment of functional MRI modalities alongside conventional morphological imaging techniques has been steadily rising in the study of head and neck cancer [52, 54]. Diffusion weighted imaging allows to infer information on tumor cellularity and ultrastructure [52, 54, 60–63], whereas Dynamic contrast-enhanced MRI (DCE-MRI) allows for the indirect evaluation of the kinetic of distribution of contrast agent and, thus, of the nature of tumoral vascularization [48, 51, 64, 65].

#### Diffusion weighted imaging (DWI)

**Fig. 6:**
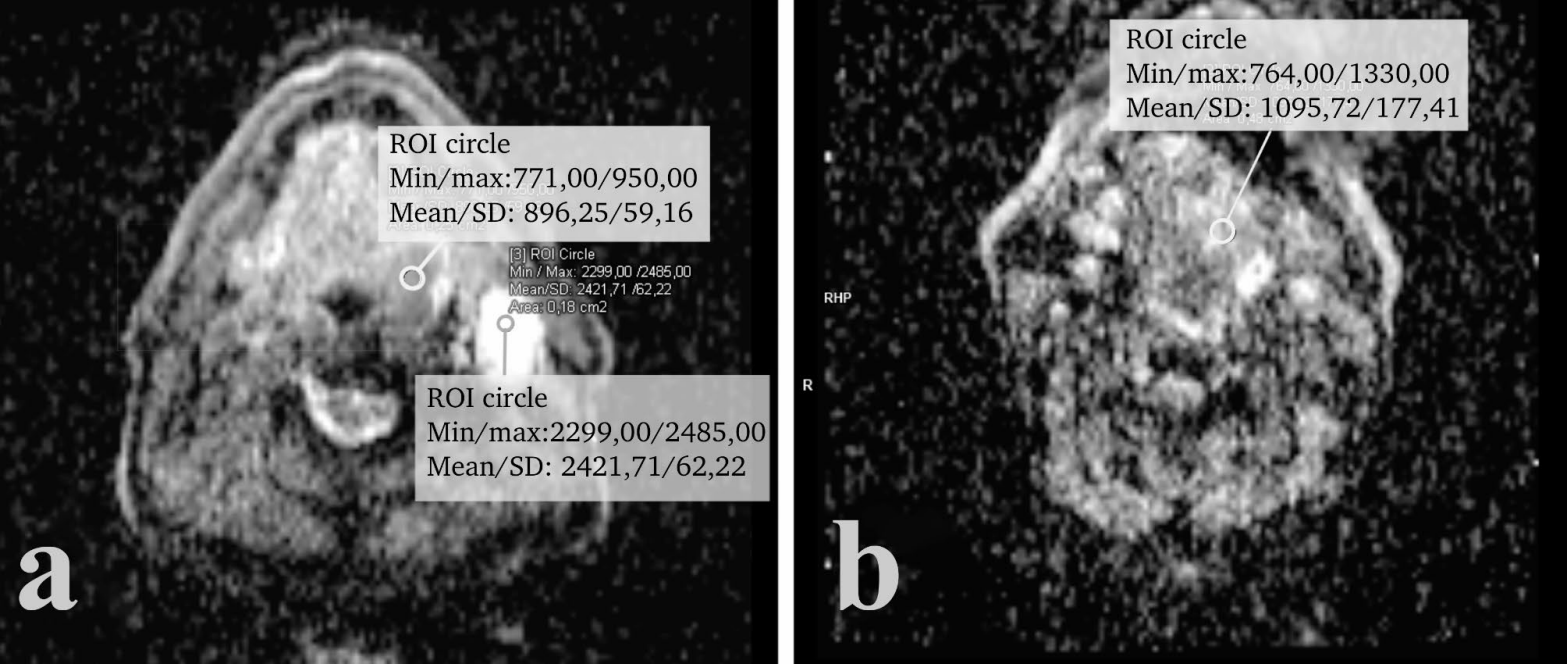
ADC map of HPV+ (a) and HPV- (b) oropharyngeal cancer. Notice the lower mean ADC value of the HPV + lesion (896,25 vs. 1095,72)

When it comes to DWI, it has been reported that HPV + lesions tend to overall show lesser values in both tissue diffusion coefficient (D_t_) and apparent diffusion coefficient (ADC) when compared to HPV- tumors [25, 48, 54, 56, 66, 67] (Fig. 6). This difference seems to stem from the different architectural heterogeneity that the two neoplasms have, with HPV- cancer showing higher microstructural cellularity [66].

It should also be noted that many authors [66, 67] have pointed out that, while most ADC maps are obtained monoexponential calculation with 2 b values (most often 0-800/1000), the choice of b value affects the ability of DWI to discern HPV status, the “optimal” choice being 0-1000 [67]. To this end, pixel-based calculation of ADC maps in like parametric maps and ADC histograms have been shown to be able to asses HPV status [66, 67]. Studies seem to suggest that HPV- cancer shows a symmetric normal distribution of ADC histograms, correlating with it microstructural heterogeneity, while HPV + cancer seems to show leptokurtic skewed right ADC histograms [66].

The lower values in DWI seem to not only be typical of primary site HPV + disease, but also for metastatic HPV + lymph nodes which show overall lower ADC and D_t_ than their HPV- counterpart [25, 54, 56].

It should also be noted that other forms of diffusion weighted imaging such as intra-voxel incoherent motion (IVIM) with evaluation of D* and diffusion kurtosis imaging (DKI) with evaluation of skewness and kurtosis don’t seem to produce any significant element of differential diagnosis for HPV status [48, 54] though some studies, such as Salzillo et al. [30], report finding that HPV + primary site tumors tend to have higher kurtosis and skewness values and lower values of D* (Table 1). Nonetheless, the value of these imaging modalities in the definition of HPV status seems to be a topic for further studies.

#### Dynamic contrast-enhanced imaging (DCE-MRI)

**Fig. 7:**
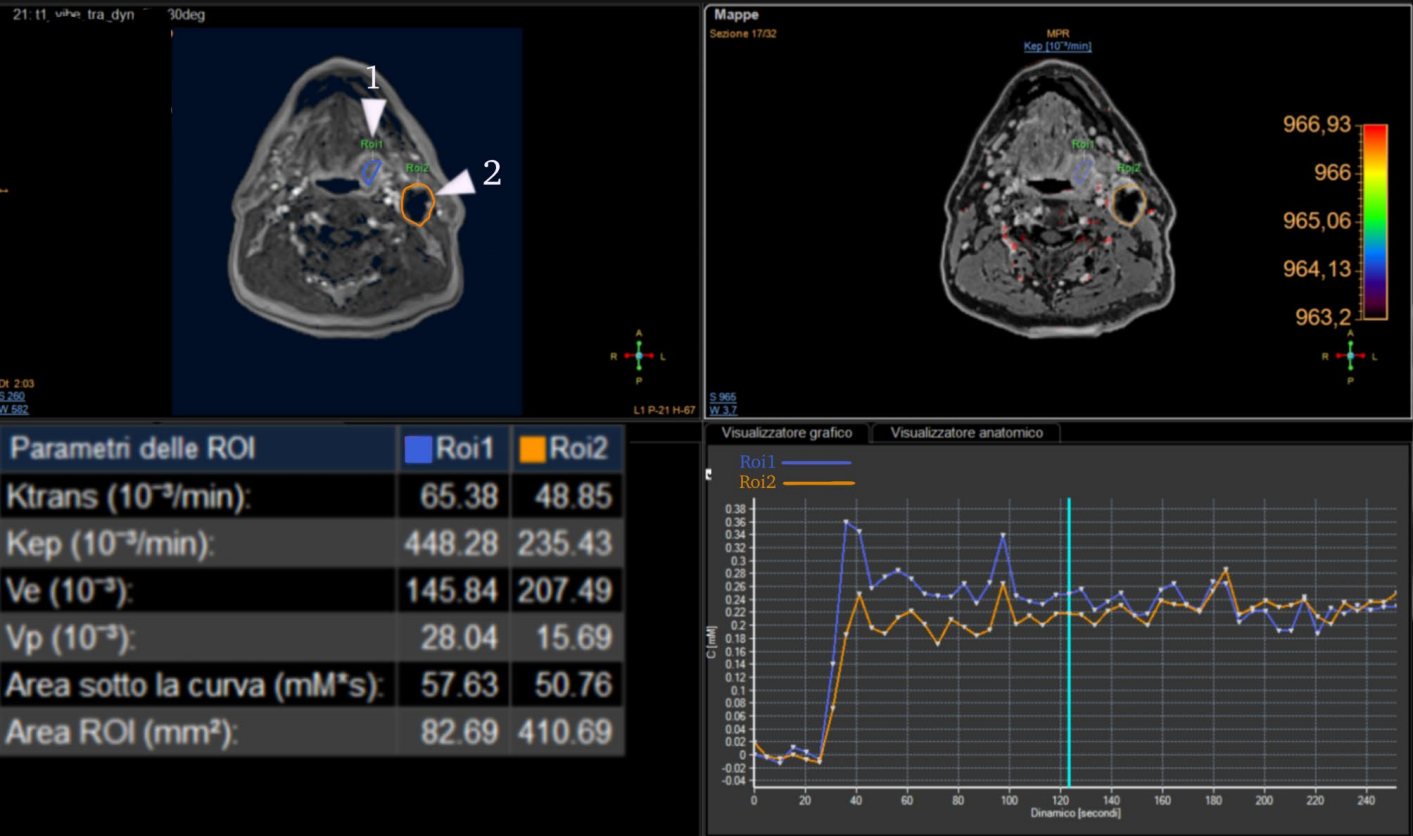
Perfusion curve of HPV + oropharyngeal cancer (1) and metastatic lymph node (2)

**Fig. 8:**
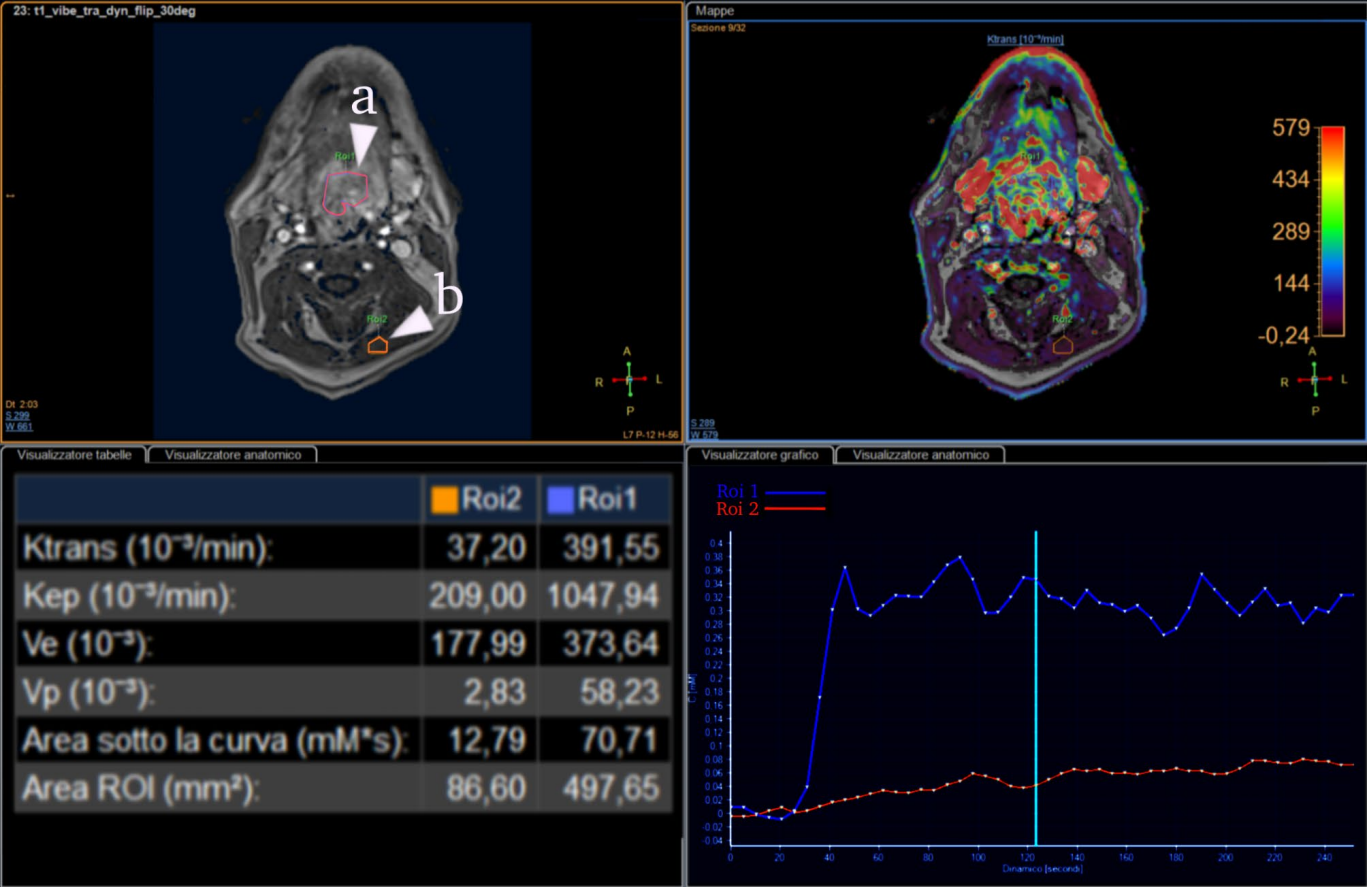
Perfusion curve of HPV- oropharyngeal cancer (a, ROI1) compared to muscle (b, ROI2). Notice the lower Ktrans value when compared to the one in Fig. 7

The role played by DCE-PWI in the seems to be unclear, though higher values of K_trans_ parameter tend to be associated with HPV + lesions, this association seems to be inconstant and not significative enough to provide sufficient evidence in most cases [30, 68–70] (Figs. 7 and 8).

## Role of texture analysis

Radiomics with texture analysis is a new technique that in recent years is gaining lot of visibility for its capacity of characterize tissue heterogeneity in order to identify and to differentiate structures such as neoplasms or organs, using a region of interest (ROI) and the extraction of features. It can be applied on CT, MRI and also on positron emission tomography (PET-CT) [71–78]. It consisted in measuring the spatial distribution of pixel values to retrieve structural information not perceptible by human eye. It is based on the extraction of first, second and higher-order parameters. The first order uses the histogram to study the gray-level distribution frequency within the ROI in order to evaluate the single pixel and not its interactions with adjacent pixels. The second order evaluates how often the intensity of one pixel has a specific relationship to that of another pixel through gray-level co-occurrence matrix (GLCM) measurements. A further way to derive second-order parameters is the gray-level run length matrix (GLRLM) that analyses consecutive pixels with the same intensity in a defined direction. The higher orders assess differences between pixels and voxels in the context of the entire ROI using a neighborhood gray-tone-difference matrix (NGTDM) by identifying variations within the examined space in gray-level intensity [79–84]. The use of texture analysis is becoming increasingly used as an important tool in support for the radiologist in the diagnosis and assessment of disease recurrence-persistence after surgery or radiochemoteraphy. It can also be used to evaluate prognosis and in particular in the early differentiation between HPV + or HPV- neoplasms and also to detect and evaluate lymph node neck metastases.

### Diagnosis

The use of radiomics applied on CT and MRI images has becoming increasingly interesting both for diagnosis and evaluation of pre-therapy HPV + oropharyngeal cancer. In fact, lot of studies are evaluating whether these tools can be able to characterize tumour histotypes [85–92] and in particular HPV + from HPV-, because of the great difference in prognosis and response to therapy of these two different neoplasms [93–96]. For this reason, several studies were conducted in order to differentiate and evaluate preliminarily the HPV status of a lesion both on CT and on MRI imaging. The first order features included shape features, both descriptors of the two or three-dimensional size and shape of the ROI. In the study by Choi et al. [86], for example, CT images of 86 untreated patients were analyzed and it was found how lower values of a specific feature (spherical disproportion) was associated to HPV positivity, due to their more regular shape. Similar results were also found in the study by Yu et al. [87]. Also, Bos et al. [97] based their study on the fact that HPV + primary tumours tend to have a more regular shape and some features can correctly assess their rounder appearance. Differently, histogram features study the gray-level distribution frequency within the ROI in order to evaluate the single pixel and not its interactions with adjacent pixels. Always on CT images, Bogowicz et al. [89], Ranjbar et al. [90], Fujita et al. [91] as also Buch at al. [92] assessed how histogram features were able to discriminate HPV status. Differently, de Perrot et al., analysed ADC-MRI sequences [66]. The higher orders assess differences between pixels and voxels in the context of the entire ROI by identifying variations within the examined space in gray-level intensity. Leijenaar at al. [88] evaluated how low-gray-level-large-size-emphasis, was higher in HPV + tumours. Regarding MRI imaging, Dang et al. [98] conducted a study on T1 and T2 weighted images and DWI sequences, with around 80% accuracy in differentiate HPV + from HPV- tumours.

### Differential diagnosis

As already said, many studies have tried to evaluate whether radiomics features are able to differentiate malignant and benign lesion or to evaluate HPV status of malignant lesions. Regarding the differentiation of the status, a study by Choi et al. [86] and the one by Yu et al. [87], demonstrate how some features, including a shape one, SphericalDisproportion, was statistically significant in differentiate HPV + from HPV- tumours, due to their less complex shape. Also, histogram features, such as median and entropy, were able to differentiate the HPV status in different studies such as the one by Fujita et al. [91], and the one by Buch et al. [92]. Features representing the tissue heterogeneity were found significant in different studies, with features of heterogeneity with lower values in HPV + tumours, related to their greater structural homogeneity [88, 89].

Radiomics has also been used to try to differentiate OPSCC from other tumours that can affect tissues like tonsils, as for example, lymphoma as in the study by Bae et al. [99] on MRI imaging, due to the grater heterogeneity of OPSCC tumour different from lymphatic tissue.

Texture analysis has been used also to discriminate normal tonsillar tissue from neoplastic one, basing the study not only on structural, morphological and dimensional parameters, but also on the heterogeneity of the structure, as in the study by Kim et al. [100].

### Prognostic evaluation and follow-up

The evaluation of prognostic features capable of assessing patients with high risk of distant metastases in HPV + OPSCC is very important in order to establish the correct treatement. In a study by Rich et al. [101] they found out some features able to separate high risk patients from low risks ones. Other important application is the evaluation of neoplasms at a high risk of non-response or recurrence after therapy. This is in particular an important tool, that in the future could be of great aid in the correct therapeutic decision-making, in order to potentially enable personalized radiotherapy treatment [102–104]; regarding this application, the study by M.D. Anderson Cancer Center, Houston, Texas, USA [105] performed on 465 patients, has tried to correlate response to therapy in OPSCC patients, with different clinical prognostic factors. The research of radiomics features capable of creating prognostic and risk model have been applied both in CT and PET/CT imaging [100, 106–109]. One of these, by Cozzi et al. [110]; assessed how some features correlate with survival and local control after RT-CHT.

### Limits

Although radiomics is not yet used in clinical practice, it may become in the future a valuable aid in the diagnosis, evaluation of HPV status and therefore aggressiveness of squamous cell tumours and, therefore, in decision-making process. There are currently limitations to the use of texture analysis. One of the challenges is the necessary implementation of standardised methods for the analysis of textural parameters, due to the large number of different procedures and software that can be used (free and non-free commercially available or custom-made-in-house applications) [111–113]. In addition, the great amount of variables during the execution of the examination, both on CT and MRI (different machines used, types of acquisition or sequences, post-processing algorithms, type of software used) also make the results difficult to reproduce at this time [114–116]. Also, the full data processing should be conducted in the shortest possible time with the possibility to analyse them by implementing the software systems where radiologists are used to visualise images. The addition of texture analysis should lead to improved diagnostic performance and in clinical practice, should be integrated with other CT, MRI and clinical parameters to better characterize the tissue investigated [106, 117].

## Conclusions

HPV-related cancer of the oropharynx is characterized by different treatment options and prognosis compared to its HPV- counterpart. It is thus of paramount importance to identify means to distinguish between the two entities. While at present biopsy is the gold standard for diagnosis, there are a plethora of imaging criteria that can help the radiologist infer the nature of a primary lesion of the oropharynx or of a metastatic lymph node. Knowledge of these criteria is fundamental for any radiologist that deals with the head and neck district and will become more and more important as HPV-related disease continues to rise in numbers.

## Data Availability

Not applicable.
